# Cancer Immunotherapy and Personalized Medicine: Emerging Technologies and Biomarker-Based Approaches

**DOI:** 10.4172/2155-9929.1000350

**Published:** 2017-06-28

**Authors:** Laura Maciejko, Munisha Smalley, Aaron Goldman

**Affiliations:** 1 Integrative Immuno-Oncology Center, Mitra Biotech Woburn, MA 01801, USA; 2 Department of Medicine, Harvard Medical School, Boston, MA 02115, USA; 3 Division of Engineering in Medicine, Department of Medicine, Brigham and Women’s Hospital, Boston, MA 02115, USA

**Keywords:** Tumor ecosystem, Cancer immunotherapy, Precision diagnostics, Biomarker

## Abstract

**Purpose of review:**

The vision and strategy for the 21st century treatment of cancer calls for a personalized approach in which therapy selection is designed for each individual patient. While genomics has led the field of personalized cancer medicine over the past several decades by connecting patient-specific DNA mutations with kinase-targeted drugs, the recent discovery that tumors evade immune surveillance has created unique challenges to personalize cancer immunotherapy. In this mini-review we will discuss how personalized medicine has evolved recently to accommodate the emerging era of cancer immunotherapy. Moreover, we will discuss novel platform technologies that have been engineered to address some of the persisting limitations.

**Recent finding:**

Beginning with early evidence in personalized medicine, we discuss how biomarker-driven approaches to predict clinical success have evolved to account for the heterogeneous tumor ecosystem. In the emerging field of cancer immunotherapy, this challenge requires the use of a novel set of tools, distinct from the classic approach of next-generation genomic sequencing-based strategies. We will introduce new techniques that seek to tailor immunotherapy by re-programming patient-autologous T-cells, and new technologies that are emerging to predict clinical efficacy by mapping infiltration of lymphocytes, and harnessing fully humanized platforms that reconstruct and interrogate immune checkpoint blockade, *ex-vivo*.

**Summary:**

While cancer immunotherapy is now leading to durable outcomes in difficult-to-treat cancers, success is highly variable. Developing novel approaches to study cancer immunotherapy, personalize treatment to each patient, and achieve greater outcomes is penultimate to developing sustainable cures in the future. Numerous techniques are now emerging to help guide treatment decisions, which go beyond simple biomarker-driven strategies, and are now we are seeking to interrogate the entirety of the dynamic tumor ecosystem.

## Introduction

According to the National Cancer Institute (NCI) there were 1,685,210 new cancer diagnoses in 2016 [1]. With such a high number of patients, effective treatment to halt the progression and/or cure cancer is of primary concern. Traditionally, cancer patients are prescribed a predetermined first line treatment depending on the cancer indication and grade. As our understanding for the complexity and uniqueness of each individual tumor improves, the suitability of the this ‘one-size-fits-all’ approach to cancer therapy is called into question. Precision medicine, the proposed future for the treatment of disease, is based on a tailored approach for selecting therapy at the individual patient level [2]. Given the high patient-to-patient variability, cancer is a prime candidate for the use of precision medicine. Indeed, it is important that the first line therapy is effective since relapse can reduce 5-year survival rates by more than 50% in aggressive cancers [3]. It is now clear that the future of personalized cancer treatment will involve a complete biochemical characterization of the tumor using multi-dimensional analyses for a range of biological endpoints, which will lead to a calculated decision on the appropriate treatment. Thus, resulting in significantly improved Overall Response (ORR) and Overall Survival (OS) rates.

The recent discovery that tumors co-opt immune check-points as a mechanism of escaping immune surveillance has led to a renaissance in immunotherapy, and revolutionized cancer treatment options [4,5]. This suggests that inherent patient-specific biology is likely driving responses [6,7] which is thought to be largely dependent on the global immune system of each individual patient, and not necessarily dependent only on the tumor biology [8]. Therefore, new technologies are needed that can help predict which patients will benefit from immunotherapy. While cancer immunotherapy has eliminated terminal disease in some patients, clinical success is highly variable [9]. Indeed, the emerging paradigm of cancer immunotherapy is bringing with it added complexity to the challenge of personalized cancer medicine, and an answer for it that has yet to be fully elucidated [6,7,10–13]. Effective implementation of personalized cancer medicine is limited by the lack of well-established preclinical models and methods that integrate global tumor heterogeneity, with high fidelity, in terms of the role of cancer and stromal cells, tumor microenvironment, 3-D architecture, and immune contexture [14]. Indeed, current ‘gold standard’ *in-vitro* and *ex-vivo* preclinical approaches that interrogate drugs using cell lines and spheroids [15–17] or *ex-vivo* organotypic tumor models are all limited by their inability to capture the full biological approximation of the native tumor, resulting in poor mapping to clinical outcomes [17–20].

Here, we will highlight some of the pervading strategies in personalized medicine, and further elucidate how research is moving beyond the “one size fits all” approach to treatment selection.

## Classic Biomarker-Based Approaches for Precision Diagnostics

Discovery of molecular cancer biomarkers (i.e., cancer ‘addictive’ oncogenes) has paved the way for the first generation of personalized therapy. Indeed, genomic screening approaches have been commonly employed to identify tumor-specific, overexpressed proteins or genetic mutations that may confer mechanisms of therapeutic resistance in cancer cells [21]. Targeting these protein biomarkers therapeutically can lead to better clinical outcomes. For example, antibody and small-molecule inhibitors of specific proteins, such as the Human Epidermal Growth Factor Receptor 2 (HER2) has led to successful implementation of diagnostic tools like HercepTest [22,23]. The discovery of HER2 overexpression in other indications has led to the approval, in 2010, of Trastuzumab for gastric or gastroesophageal junction adenocarcinoma [24]. Interestingly, the FDA-approval of existing therapies for new indications is common, with the repurposing of thalidomide to treat multiple myeloma being a notable example [25,26].

Similarly, the overexpression of EGFR in various types of cancer was discovered as early as 1997 [27]. This led to the development of specific EGFR tyrosine kinase inhibitors (EGFR-TKIs) such as gefitinib or afatinib. However, acquired resistance to these therapies occurs in approximately 60% of patients treated with first line EGFR-TKIs [28]. It has subsequently been found that a specific mutation of EGFR is responsible for this acquired resistance, T790M. This mutation has also been observed in other types of EGFR-TKI-resistant lung cancers. The recently FDA approved small molecule inhibitor, osimertinib (Tagrisso), is an EGRF-TKI and is most effective in patients with this acquired EGFR mutation, and is used following failure of first line EGFR-TKI therapies [29]. More recently, midostaurin (Rydapt), a small molecule inhibitor of VEGF, has been approved for use in acute myeloid leukemia (AML) patients with mutated FMS-like tyrosine kinase inhibitor 3 (FLT3) [30,31]. With only 10% of leukemia patients and 25% of AML patients presenting with mutations in FLT3, biomarkers are becoming increasingly useful to connect patients to the correct therapy [32]. As one example, the companion diagnostic LeukoStrat CDx FLT3 mutation assay, was FDA-approved alongside midostaurin in April 2017, and the Phase III trial showed an improved OS rate of 23% [33].

While the biomarker-driven approach for treatment decision making in the clinic usually yields better than standard of care results, with an improvement on ORR and OS rates, there are currently only 16 drugs out of more than 200 FDA-approved agents that require, or benefit from a companion diagnostic test [34]. Moreover, the emerging generation of new anticancer therapies, which seek to invigorate the body’s own immune system, suggests that new approaches for personalized medicine must evolve.

## Targeting the Immune Compartment

### Cytokine stimulation

William Coley, commonly referred to as one of the pioneers of cancer immunotherapy, recognized the link between a patient’s complete remission from sarcoma with a *Streptococcus pyogenes* infection in 1890 [35]. This led to him treating cancer patients with bacteria post-surgery, resulting in an immune response that would keep any tumor resurgence at bay. Although this approach was largely unsuccessful due to the adverse effects of the infection, it demonstrated that activating the immune response could be of therapeutic benefit in cancer. It was not until 1992 that the first immunotherapy was approved by the FDA; high dose interleukin-2 (HD IL-2), for renal cell carcinoma (RCC) [36]. From 1986 to 2006, the NCI treated 259 metastatic RCC patients with HD IL-2. The objective response rate was just 20%, with 23 patients achieving a complete response and 30 patients achieving a partial response [36]. A similar response rate was observed with HD IL-2 treatment in melanoma, which was FDA-approved in 1998 [37]. This non-specific immunotherapeutic approach is one of very few therapies that can result in complete remission, albeit in a small cohort of patients.

### Immune checkpoint inhibitors

Immune checkpoints are negative regulators of T cell immunity, usually inhibiting the stimulation of T cells. The primary function of immune checkpoint inhibition is to rescue T cells from exhaustion or deplete T regulatory cells (T_reg_). Indeed, the best characterized immune cells in cancer biology are CD4^+^ helper T-cells, which exacerbate tumor proliferation, and cytotoxic CD8^+^ T-cells, which have been shown to prevent tumor growth [38]. One of the many receptors involved in immune checkpoints, cytotoxic T-lymphocyte-associated protein 4 (CTLA-4), discovered in 1987, modulates the extent of T cell activation by competitively binding to B7 proteins, which are required for stimulation of T cells. However, it was not until 1996 that targeting CTLA-4 was shown to have anti-cancer effects in mice [39]. This seminal study led to the development of the first immune checkpoint inhibitor, ipilimumab (Yervoy), an anti-CTLA-4 monoclonal antibody, which was approved in 2011 for metastatic melanoma. The success of ipilimumab led to the development of even more efficacious immune checkpoint inhibitors such as pembrolizumab (Keytruda) and nivolumab (Opdivo), PD-1 inhibitors, and atezolizumab (Tecentriq) a PD-L1 inhibitor. Merck’s pembrolizumab has been particularly successful, being FDA-approved for a range of indications, including metastatic melanoma, metastatic non-small cell lung cancer (NSCLC) and refractory Hodgkin’s Lymphoma. A recent report of a phase III trial showed 10.3 months progression free survival for untreated advanced NSCLC treated with pembrolizumab, compared with 6 months for platinum-based chemotherapy [40]. Moreover, in May 2017, the FDA approved pembrolizumab for the treatment of solid tumors found to be microsatellite instability-high or mismatch repair deficient. This is the first time the FDA has approved a therapy based on a biomarker rather than the location of the cancer, exemplifying the progression of personalized medicine. With 734 on-going clinical trials for immunotherapies combined with other therapies, the clinical potential of immune checkpoint inhibitors is yet to be fully realized.

### Adoptive cell transfer: Personalized T-cell therapy

Chimeric antigen receptor (CAR)-T cells are an example of adoptive cell transfer (ACT). CAR T cells taken from a patient (or other human) are engineered to express cancer-specific antigens *ex vivo* and are administered back into the patient. CAR-T cell therapy has shown efficacy in the treatment of many B cell malignancies, most notably against B cell acute lymphoblastic leukemia (B-ALL). In a study with 53 children and young adults with CD19^+^ ALL, 50 out of 53 patients went into complete remission following treatment with CAR-T cells [41]. Despite these incredible results in liquid cancers, treatment using CAR-T cells is more difficult in solid tumors, perhaps due to physical and biochemical differences. In a phase I clinical study, patients with EGFR-positive relapsed/refractory NSCLC were treated with EGFR-targeted CAR-T cells. Of 11 evaluable patients, only two patients showed a partial response and five had stable disease for two to eight months [42]. There have been proposed theories to further improve CAR-T cell therapy such as enhancing the selectivity of the CAR and using this therapy in conjunction with immune checkpoint inhibitors [43]. Whilst CAR-T cell therapy is very promising, there are many noted negative side effects, including the potentially fatal cytokine release syndrome, which also have to be addressed if this therapy is to be adopted for wider use.

Another example of ACT in the clinic is the expansion of patient tumor infiltrating lymphocytes (TIL) with IL-2, *ex vivo*, with subsequent administration directly into the tumor. This approach has resulted in promising OS rates in the clinic. It has been used to treat B cell malignancies and melanoma with great success in a small subset of patients. Moreover, in a recent study, 3 out of 9 of metastatic cervical cancer patients treated with human papillomavirus targeted-TIL responded; 2 of which experienced complete regression lasting up to 22 months [44].

## Biomarkers for Personalized Cancer Immunotherapy

While tumor associated biomarkers have been employed to personalize kinase-targeted agents for each patient (as described in more detail above), these same approaches have been less robust for personalized cancer immunotherapy. Given the complexity of the tumor microenvironment, and the heterogeneous cellular landscape, there is a clear need to improve upon our classic biomarker-based approaches to incorporate the entirety of the tumor ecosystem, and tumor-immune contexture [7,45].

In the context of the immune system, the ability for T-cells to mount a response to invasive growth of tumors is associated with improved outcome to therapy [46]. Several studies have now established that spatial distribution and infiltration of lymphocytes into tumors can predict progression-free or overall survival [47]. Indeed, lymphocytes of two distinct subclasses (CD3^+^/CD8^+^, CD3^+^/CD45RO^+^, or CD8^+^/CD45RO^+^) will confer distinct infiltration patterns that map to distinct predictive outcomes [47]. Halio Dx, a French cancer diagnostics company, has created the first standardized immune-based assay Immunoscore^®^ Colon for quantifying spatial heterogeneity of infiltrating immune cells, Immunoscore^®^ Colon [48]. Specific for colon cancer, the test requires a tumor core and invasive margin, which are studied to determine the distribution and density of both CD3^+^ T lymphocytes and CD8^+^ cytotoxic T cells. An algorithm is used to generate their Immunoscore^®^ (I4 indicating the most cytotoxic and memory immune infiltrate and I0 indicating the lowest) [48]. Groups of patients were defined based on microsatellite status and Immunoscore and then analyzed for survival. Regardless of microsatellite status, patients with an Immunoscore of I3 and I4 had prolonged disease-specific survival (DSS) and OS. In addition, patients with a score between I0–I2 showed higher risk of relapse, shorter DSS and OS [49]. This approach, investigating the number and density of infiltrating lymphocytes, has clearly shown to be beneficial and better than existing measures of response, it is important to note that the immune marker expression profile of immune cells can be a valuable predictor of response. For instance, in clinical trials using ipilimumab, melanoma patients with higher expression of genes involved in immune function, including CD8A, CD27, CD38, CD3, CD40, GZMB, PRF1, CCL4, were more likely to have positive response to treatment [50]. Despite these advances, evidence for dynamic mechanisms of resistance have led to new speculation about how to personalize cancer immunotherapy [4,10].

**Figure 1:**
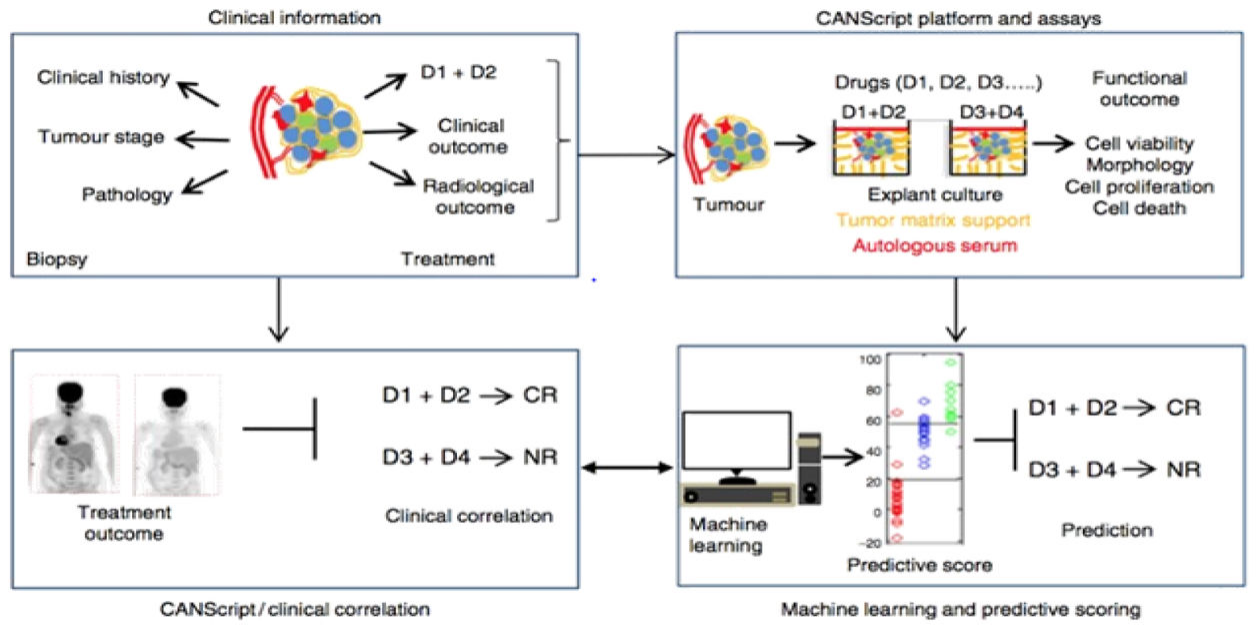
CANscript® platform technology. Four critical modules were integrated in generating and validating the CANscript platform. The first module involved collecting tumor core or surgical biopsy with tumor staging and/or pathology information besides clinical history. In the second module, tumor biopsy was rapidly processed into thin explants. The explants were cultured with tumor- and grade-matched TMPs and autologous serum (AS) and incubated with selected drug regimens. While multiple drug regimens can be used, the one used by the oncologist for the patient was always included in the tumor explant culture. The *in vitro* functional outcome of treatment in terms of cell viability, pathological and morphological analysis, cell proliferation, and cell death was quantified. In module three, these quantitative scores from the explants were aggregated using a machine learning algorithm to assign a final score, which helped rank the outcomes as complete response (CR), partial response (PR), or no response (NR). In the final module, these predictions were tested against clinical outcomes. D1, D2, D3, and D4 indicate different drug regimens (image courtesy of Mitra RxDx).

Mitra Mitra RxDx Inc., a personalized cancer medicine company based in Boston, Massachusetts, created an *ex vivo* tumor model, which harnesses fresh tumor biopsies and surgical excisions to study the effect of drugs in a fully humanized, autologous context, termed CANscript^®^ [51]. CANscript^®^ preserves the cellular and microenvironment heterogeneity by maintaining the tumor tissue in the presence of autologous plasma and peripheral blood nucleated cells (PBNC) in tissue culture wells coated with cancer type and grade-matched tumor matrix proteins (TMP). These elements enable accurate re-capitulation of the 3D architecture of the tumor and the tumor microenvironment, including the immune compartment. This *ex-vivo* platform has been used to evaluate the functional effects of conventional cytotoxic and kinase targeted cancer therapies. Using a series of endpoints following drug exposure. This was used to train a machine learning algorithm which generates an ‘M-score’ for each drug tested, which is predictive for drug response (Figure 1).

Excitingly, this platform demonstrates the first use for dynamic cellular heterogeneity, and real-time analysis of the tumor-immune contexture under pressure of immune checkpoint inhibitors. For example, Mitra recently demonstrated the flexibility of the platform to the assay to capture changes to the T-cell immune repertoire, and help predict the clinical efficacy of multiple immuno-oncology agents, such as immune checkpoint inhibitors [52,53]. Indeed, by mapping the role of T-regulatory (T_reg_) cells in the presence or absence of multiple PD-1 checkpoint blockade, their research team elucidated that antitumor outcomes can be unique within a single patient sample. They discovered that depletion of T_reg_, but not increase of IFN-g expressing cytotoxic T-cells correlates to antitumor response. These findings are the first to support patient-specific response to a single immunotherapy among multiple drugs that target the same immune checkpoint. Such revelations could help shape the future of treatment selection in immunotherapy at the individual patient level.

## Concluding Remarks

Cancer treatment decision-making is the final frontier in our challenge to cure cancer. While biomarkers, oncogenes and mutations have been classic maps for predicting therapy outcome, clinical success has reached a plateau. Even in the new age of cancer immunotherapy, target-specific molecular characteristics of a tumor are only present in a small proportion of patients. Immunotherapies can also cause immune-related adverse events, especially with CTLA-4, which can be serious and affect a large proportion of treated patients, with one trial observing it in one third of patients [54]. This makes prior patient testing even more vital to ensure these powerful next generation treatments are prescribed to the correct population.

The current problem is that the mechanism by which a drug target is selected is dictated by our knowledge of the role of growth factor receptors or key immune checkpoint proteins, and not patient-specific biology. There are a multitude of overexpressed, mutated and/or dysfunctional proteins in a tumor. Targeting one may not yield the best response rates for patients. There needs to be a way of deciding the relative efficacy of multiple treatment options for patients, in order to be sure of giving them the best possible chance of surviving and overcoming their cancer. We are in the midst of an exciting time for the development of truly personalized medicine, with the emergence of technologies that have the capacity to predict clinical outcomes. A more unbiased approach to drug selection has been sought with the invention of high throughput sequencing, searching the whole genome for mutations, but there is still, a disconnect between the discovery of mutations and deciding which drug is administered. The discovery of new platform technologies, which go beyond molecular biomarkers by seeking to interrogate the dynamic and heterogeneous microenvironment which go beyond characterizing individual biomarkers may lead to improved personalized approaches for cancer immunotherapy. Not only do these types of platforms ensure that the patient gets the best treatment for their specific type of cancer, but it also paves the way for the discovery of drug efficacy in tumors that might not otherwise have been tested, leading to new drug discovery or repurposing of immunotherapy with conventional drugs.
